# Selective cognitive and psychiatric manifestations in Wolfram Syndrome

**DOI:** 10.1186/s13023-015-0282-1

**Published:** 2015-05-30

**Authors:** Allison N. Bischoff, Angela M. Reiersen, Anna Buttlaire, Amal Al-lozi, Tasha Doty, Bess A. Marshall, Tamara Hershey, Washington University Wolfram Syndrome Research Group

**Affiliations:** Department of Psychiatry, Washington University School of Medicine, Campus Box 8225, 4525 Scott Avenue, 63110 St. Louis, MO USA; Department of Pediatrics, Washington University School of Medicine, St. Louis, MO USA; Department of Cell Biology, Washington University School of Medicine, St. Louis, MO USA; Department of Neurology, Washington University School of Medicine, St. Louis, MO USA; Department of Radiology, Washington University School of Medicine, St. Louis, MO USA

**Keywords:** Wolfram syndrome, Cognition, Psychiatry, Behavior, Development, Diabetes mellitus

## Abstract

**Background:**

Wolfram Syndrome (WFS) is known to involve diabetes mellitus, diabetes insipidus, optic nerve atrophy, vision loss, hearing impairment, motor abnormalities, and neurodegeneration, but has been less clearly linked to cognitive, sleep, and psychiatric abnormalities. We sought to determine whether these abnormalities are present in children, adolescents, and young adults with WFS compared to age- and gender-matched individuals with and without type 1 diabetes using standardized measures.

**Methods:**

Individuals with genetically-confirmed WFS (*n* = 19, ages 7–27) were compared to age- and gender- equivalent groups of individuals with type 1 diabetes (T1DM; *n* = 25), and non-diabetic healthy controls (HC: *n* = 25). Cognitive performance across multiple domains (verbal intelligence, spatial reasoning, memory, attention, smell identification) was assessed using standardized tests. Standardized self- and parent-report questionnaires on psychiatric symptoms and sleep disturbances were acquired from all groups and an unstructured psychiatric interview was performed within only the WFS group.

**Results:**

The three groups were similar demographically (age, gender, ethnicity, parental IQ). WFS and T1DM had similar duration of diabetes but T1DM had higher Hb_A1C_ levels than WFS and as expected both groups had higher levels than HC. The WFS group was impaired on smell identification and reported sleep quality, but was not impaired in any other cognitive or self-reported psychiatric domain. In fact, the WFS group performed better than the other two groups on selected memory and attention tasks. However, based upon a clinical evaluation of only WFS patients, we found that psychiatric and behavioral problems were present and consisted primarily of anxiety and hypersomnolence.

**Conclusions:**

This study found that cognitive performance and psychological health were relatively preserved WFS patients, while smell and sleep abnormalities manifested in many of the WFS patients. These findings contradict past case and retrospective reports indicating significant cognitive and psychiatric impairment in WFS. While many of these patients were diagnosed with anxiety and hypersomnolence, self-reported measures of psychiatric symptoms indicated that the symptoms were not of grave concern to the patients. It may be that cognitive and psychiatric issues become more prominent later in life and/or in later stages of the disease, but this requires standardized assessment and larger samples to determine. In the relatively early stages of WFS, smell and sleep-related symptoms may be useful biomarkers of disease and should be monitored longitudinally to determine if they are good markers of progression as well.

**Trial Registration:**

Current Clinicaltrials.gov Trial NCT02455414.

## Background

Wolfram Syndrome (WFS) (OMIM #222300) [1] is a rare autosomal recessive disease typically characterized by diabetes mellitus, diabetes insipidus, optic nerve atrophy, hearing and vision loss, motor impairment, neurodegeneration, and a reduced lifespan. WFS can be caused by mutations in the *WFS1* gene, which is known to encode the wolframin protein. Wolframin exists within the endoplasmic reticulum (ER) membrane [2] and helps to protect cells from ER stress related apoptosis [3–5]. Wolframin deficiency leads to cell death in the insulin producing β-cells in the pancreas, causing diabetes, and is also thought to underlie cell death in the brain [6]. As cellular studies move towards possible clinical interventions [7], an understanding of the natural history of WFS is becoming more critical, and the progressive neurological aspects of WFS, including brain structure abnormalities, gait impairment, and cognitive and behavioral difficulties, are receiving increasing attention [8–10].

Recent work has shown that altered brain structure in the brainstem and cerebellum can be detected early in the disease process through in vivo neuroimaging [8]. Clinical retrospective data and case studies have suggested that neurological symptoms, such as ataxia and cognitive changes, occur from teenage years into mid-adulthood [11, 12], yet our direct measurements [9] found that motor neurological abnormalities, such as poor balance and altered gait, are present in childhood and early adolescence. It is unknown at this point whether non-motor, complex, and higher order neurological functions, such as cognition and emotional functions, also follow this pattern, or if those functions are relatively spared early in the disease due to their independence from brainstem and cerebellum function. However, these domains have been poorly quantified in previous studies. While several case studies reported cognitive impairment and depression in adult patients with advanced WFS [12–16], others described patients with normal intelligence [17, 18]. Studies typically have lacked standardized testing or an age-matched control group for comparison.

Our group has studied a cohort of children, adolescents, and young adults with WFS using standardized and quantified assessments of cognitive, psychiatric, and behavioral abnormalities. We also acquired overlapping measures on age- and gender-matched individuals with and without type 1 diabetes mellitus (T1DM). The goal of this paper is to describe the nature and extent of non-motor abnormalities in a deeply phenotyped cohort of young patients with genetically-confirmed WFS. This information may reveal biomarkers for disease progression, targets for symptomatic treatments, and potential outcome measures for future clinical trials.

## Methods

### Participants

#### WFS

WFS patients were identified through the Washington University International Wolfram Syndrome Registry and were participants in a longitudinal natural history study (Washington University Wolfram Syndrome Research Clinic) involving annual data collection over 5 years. For enrollment in the Research Clinic, patients had to be age 30 or younger and have genetically confirmed WFS (mutations of the *WFS1* gene), or diabetes mellitus and optic atrophy diagnoses before age 18. Subsets of these data have been analyzed to answer other questions [8, 10]. For this paper, we analyzed data from the 2013 clinic because that year’s clinic used a wider variety of cognitive, psychiatric, and behavioral measures and had a larger and more diverse patient sample than previous years.

#### Controls

The comparison groups consisted of individuals with type 1 diabetes (T1DM) and non-diabetic healthy controls (HC). The T1DM group was recruited through Washington University’s Pediatric Diabetes Clinic at St. Louis Children’s Hospital and Washington University School of Medicine in St. Louis. All T1DM were clinically diagnosed with T1DM based on disease presentation, physical exam, medical history, and family history. T2DM and monogenic DM were ruled out based on physical exam and personal and family history based on the guidelines established by the American Diabetes Association. The HC group was recruited through the community, or were siblings of participants in the T1DM group. Exclusion criteria included self-reported psychiatric or neurological diagnoses, the use of psychoactive medications, contraindication to MRI, and <36 week gestation with respirator use or other complications.

The study was approved by the Human Research Protection Office at Washington University in St. Louis. Informed consent was obtained prior to testing for all participants. For children under age 18, parents/guardians provided written consent and children assented to testing.

### Assessments

WFS patients performed cognitive, psychiatric, smell, and behavioral assessments, including parent-report of symptoms. In addition, Hb_A1c_ and capillary glucose levels were measured and a medication log was acquired. These measures and the resulting data are presented in this paper. WFS patients also performed vision, auditory, neurological, taste, endocrinologic, and urologic assessments (data not presented). All tests were performed over a 3 day period in July 2013. Controls performed all of the tests described below, with the exception of the WURS and an unstructured psychiatric interview, on a single day, spread throughout two years.

#### WURS

A neurologist completed the Wolfram Unified Rating Scale (WURS) [19] during the clinic on each WFS patient. The WURS was designed to assess overall disease progression and severity of physical and behavioral issues previously observed in WFS (e.g. vision, hearing, motor, urological, neurological, psychological and mood problems) and has been shown to have good inter-rater reliability and validity [19]. The Total WURS score is made up of Physical (maximum score = 160) and Behavioral (maximum score = 54) subscales.

#### Cognitive testing

Capillary glucose levels were checked prior to testing for all subjects; testing proceeded once levels were determined to be between 70 and 300 mg/dl.

1. Wechsler Abbreviated Scale of Intelligence Vocabulary and Similarities subtests (ages 6+) [20]: These subtests were used to generate a verbal intelligence quotient (VIQ).
2. Woodcock-Johnson III Spatial Relations (SR) subtest (all ages) [21] was used to assess spatial reasoning; scaled scores were generated.
3. Letter-Number Sequencing (LNS) and Digit Span (DS) subtests were used to measure short-term/working memory. These subtests came from the Wechsler Intelligence Scale for Children (ages 6–15) [22] or the Wechsler Adult Intelligence Scale (16+) [23] and generated scaled scores.
4. California Verbal Learning Test (CVLT-C for ages 0–15 [24] and CVLT-II for ages 16+ [25]) was used to assess verbal learning and delayed memory. Short-term and long-term delay scaled scores were used in analyses.
5. Conner’s Continuous Performance Task (CPT) (ages 6+) [26] is a computerized assessment of sustained attention and impulsivity. Hit Reaction Time and Detectability scaled scores were used in analyses.
6. University of Pennsylvania’s Smell Identification Test (UPSIT) (ages 5+) [27] was used to measure smell identification skills and raw scores were calculated.
7. Wechsler Test of Adult Reading (WTAR) [28] was used to measure a parent’s verbal intelligence and scaled scores were generated.

#### Psychiatric and behavioral interview and measures

WFS patients and their parents, as appropriate, met with a study psychiatrist to discuss current psychoactive medication use, past diagnoses, and psychiatric questionnaires completed for the clinic. The study psychiatrist, based upon past report and clinical impression, assigned a “best estimate diagnosis” to each patient. The WFS group and control groups and parents/caregivers of subjects under age 18 completed psychiatric and behavioral questionnaires.

1. Child and Adolescent Symptom Inventory 4R (CASI-4R) (given to WFS of all ages and controls ages 5–17) [29] is a parent-reported inventory that itemizes DSM-IV relevant symptoms of behavioral and emotional disorders.
2. Adult Self-Report Inventory-4 (ASRI-4) (ages 18+) [30] and Youth Inventory-4 (YI-4R) (ages 12–17) [31] are adult- and youth-reported inventories that itemize DSM-IV relevant symptoms of behavioral and emotional disorders. These inventories generate scores for a variety of symptom categories that can be assessed using the Symptom Count Score method. The Symptom Count Score method evaluates symptom frequency (never, sometimes, often, and very often) and determines if the number of symptoms above a threshold frequency meets the symptom criterion for a specific disorder. We focus here on the symptom categories that were the same across ages and reports (parent, adult, youth), namely: ADHD-Inattentive, ADHD-Hyperactive-impulsive, Conduct Disorder, Oppositional Defiant Disorder (ODD), General Anxiety, Social Phobia, Specific Phobia, Obsessions, Compulsions, Major Depressive Episode (MDE), Manic episodes (Bipolar Disorder), and Dysthymia. To note, for the Specific Phobia, Obsessions, and Compulsions categories, the CASI-4R parent-report requires a lower frequency of symptoms to meet criterion compared to the self-reported inventories (i.e., reported frequency must be “sometimes”, rather than “often” or “very often”). Furthermore, only 1 question was included in the criterion score for these categories, whereas other categories had multiple questions within the criterion score. Meeting symptom criterion cutoff did not imply a diagnosis (other criteria such as age-of-onset, duration, impairment, and exclusion criteria were not fully assessed by the instrument), but indicated a particular subject endorsed experiencing a threshold number of symptoms that may indicate a particular diagnosis.
3. Pediatric Sleep Questionnaire (PSQ) (given to WFS of all ages and controls ages 0–17) [32] and Pittsburgh Sleep Quality Index (PSQI) (ages 18+) [33] were parent- and adult self-reports used to evaluate sleep dysfunction. A raw score above 0.33 on the PSQ and a raw score above 5 on the PSQI suggest clinically diagnosable sleep problems.

### Statistical analysis

All of the data was managed using REDCap, a web-based electronic database hosted by the Biostatistics Division of Washington University School of Medicine [34]. Data was analyzed using the statistical program SPSS©.

#### Participant characteristics

Age, gender, diabetes duration, Hb_A1c_, and glucose levels were compared across groups (WFS, T1DM, HC) using univariate general linear models (GLMs) or chi-square tests. Significant main effects of group (*p* < 0.05) were followed by post-hoc comparisons.

#### Cognitive tests

Cognitive variables were compared across groups using GLMs. All cognitive variables except for the UPSIT raw scores were scaled scores, t-scores, or z-scores that provided adjustment for age and, in a subset of variables, for gender. For those scores that did not correct for gender (WTAR, VIQ, LNS, DS), gender was co-varied in analyses. UPSIT raw scores were co-varied for both age and gender. UPSIT percentile scores were not used for analysis due to limited available normative data in young ages. Significant main effects of group (*p* < 0.05) were followed by post-hoc comparisons.

#### Psychiatric and behavioral measures

For the psychiatric inventories, we focused our analysis on three major domains (Neuro-developmental and Disruptive Behavior, Anxiety Disorders, and Mood Disorders) that comprised the symptom categories previously mentioned. Neuro-developmental and Disruptive Behavior included ADHD-Inattentive, ADHD-Hyperactive-impulsive, Conduct Disorder, and ODD. Anxiety Disorders included General Anxiety, Social Phobia, Specific Phobia, Obsessions, and Compulsions. Mood Disorders included Major Depressive Episode, Manic Episodes (Bipolar Disorder), and Dysthymia. We combined overlapping adult-reported and youth-reported domain scores into a ‘self-report’ domain to reduce comparisons and increase sample size. We scored 12-year-olds in the 12–18 year range rather than the 5–12 year range. Chi-square tests were used to compare the proportion of individuals that reached the symptom criterion cutoff for one or more symptom category within each domain. Parent-report and self-report cutoffs were separately determined. PSQ scores were compared across groups using GLMs, co-varied for age and gender. PSQI scores were not used for analysis due to small sample size of adult participants. Significant main effects of group (*p* < 0.05) were followed by post-hoc comparisons.

#### Correlations

Pearson or Spearman correlations were performed between variables that were significantly impaired in the WFS group compared to controls and indictors of WFS disease severity, such as WURS scores and diabetes duration.

## Results

### Participant characteristics

We evaluated 19 genetically-confirmed WFS patients, 25 individuals with T1DM and 25 individuals without T1DM. Demographic data are shown in Table 1. Eight WFS participants had participated in the clinic for 3 previous years; 3 had participated in 2 previous years; 4 had participated in 1 previous year and 4 were participating in the clinic for the first time. Four WFS patients were non-English speaking and only completed the spatial reasoning and smell identification tasks. All participants in the control groups were native English speakers. Two WFS patients were severely vision impaired and could not complete the SR or CPT tasks. One WFS patient was severely hearing impaired and could not complete the WASI, LNS, DS, or CVLT tasks. Two other WFS patients were assessed, but their data were excluded for analysis. One patient experienced a neurological trauma (anoxic event) in early childhood that may have affected cognition, and the other was a very young, non-English speaking patient who was unable to complete cognitive tasks.

**Table 1:** Participant characteristics

	WFS	T1DM	HC	
N	19	25	25	
Gender distribution	13 F, 6 M	15 F, 10 M	10 F, 15 M	
Ethnicity distribution (%)	84.2 C, 10.5 H, 5.3 O	92 C, 4 AA, 4 H	72 C, 8 AA, 8 H, 12 O	
	Mean (SD)			p
Age	15.8 (6.0)	13.6 (4.6)	14.3 (5.6)	0.43
Diabetes duration	10.2 (5.9)	7.6 (5.0)	–--	0.13
Hb_A1c_ (%)	7.5 (1.2)^b^	8.3 (1.0)^a,b^	5.3 (0.3)	<0.001*
Capillary glucose (mg/dl)	196.5 (60.5) ^b^	209.5 (73.2)^b^	93.5 (11.8)	<0.001*

*C* Caucasian, *AA* African American, *H* Hispanic, *O* other

*significant at *p* < 0.05; *p* values shown for the main effect of group in univariate GLM analyses for each measure

^a^mean different from WFS group

^b^mean different from HC group

The WFS and control groups did not differ in age (F_2, 66_ = 0.86, *p* = 0.43), gender (*χ*^2^ (*N* = 69) = 3.91, *p* = 0.14), or diabetes duration (T1DM vs WFS; F_1, 41_ = 2.40, *p* = 0.13), even when subsamples (e.g. for variables with some missing data) were considered. However, there was a main effect of group on Hb_A1c_ (F_2, 62_ = 68.43, *p* < 0.001), with all three groups differing from each other (*p* < 0.01). There was also a main effect of group on glucose levels (F_2, 64_ = 32.57, *p* < 0.001), with HC having lower levels than both WFS (*p* < 0.001) and T1DM (*p* < 0.001); WFS and T1DM did not differ from each other. However, no glucose level was obtained at time of testing for 2 WFS patients. One of these patients does not have diabetes (Fasting glucose = 86 mg/dl). See Table 1.

### WFS clinical characteristics

Table 2 reports age of diabetes mellitus onset, age of optic atrophy diagnosis, and WFS1 allele mutations. Age of optic atrophy diagnosis may not reflect true age of onset. Eighteen WFS patients had insulin dependent diabetes mellitus and 15 had diabetes insipidus. Eighteen WFS patients had optic atrophy and 12 had hearing loss. WFS patients’ WURS Physical total scores ranged from 0 to 33 (*M* = 10.4, SD = 8.8). WURS Behavioral total scores ranged from 0 to 15 (*M* = 5.8, SD = 4.7). WURS Total scores ranged from 5 to 44 (*M* = 15.8, SD = 11.6).

**Table 2:** WFS clinical characteristics and WFS 1 clinical mutations

Age of DM onset	Age of OA diagnosis	WFS mutation allele 1	WFS mutation allele 2
2	12	c.1112G>A; p.W371X	c.1885C>T; p.R629W
3	7	c.439delC, p.R147fsX163	c.1620G>A, p.W540X
3	8	c.1230_1233delCTCT, p.Val412fsX440	c.1243_1245delGTC, p.Val415del
3	7	c.2002C>T; p.Q668X	c.2002C>T; p.Q668X
4	10	c.739_740delTT, p.Phe247fsX251	c.1243_1245delGTC, p.Val415del
5	10	c.1251_1252delCTinsG; p.Phe417Leufsx25	c.1885C>T; p.Arg629Trp
5	17	c.739_740delTT, p.F247Cfs*5	c.1243_1245delGTC,p.V415del
5	15	c.599 T>C; p.L200P	c.695G>C; p.R232P
5	5	c.599delT; p.L200fs286Stop	c.2254G>T; pE752Stop
5	6	c.1230_1233delCTCT; p.Val412fs440Stop	c.1243_1245delGTC: p.Val415del
5	No dx	c.739_740delTT, p.F247Cfs*5	c.1243_1245delGTC,p.V415del
6	7	c.817G>T; P.E273X	c.1839G>A; p.W613X
6	9	c.2648del4; p.F883fs	None identified
7	7	c.320G>A; p.G107E	c.1882C>T; p.R629W
7	8	c.376G>A; p.A126T	c.1838G>A;p.W613X
7	7	c.376G>A; p.A126T	c.1838G>A;p.W613X
13	13	c.1240_1242delTTC; p.F414del	c.1689_1694delCTTCCT; p.F564del;p.L565del
14	12	c.605A>G; p.E202G	c.631G>A; p.D211N
No dx	5	c.2339G>C, p.Gly780Ala	c.2452C>T, p.Arg818Cys

*DM* diabetes mellitus, *OA* optic atrophy, *No dx* No diagnosis in 2013

### Cognitive assessments

Table 3 reports group means and standard errors for each cognitive variable analyzed.

**Table 3:** Cognitive assessment adjusted means (±S.E.)

	Normative average	WFS		T1DM		HC		*p*
		*N*	*M*	*N*	*M*	*N*	*M*	
WTAR scaled score^c^	100	14	104.1 ± 2.5	21	105.3 ± 2.0	18	109.2 ± 2.2	0.28
VIQ^c^	100	14	111.8 ± 3.5	25	108.2 ± 2.6	24	108.8 ± 2.7	0.70
Spatial relations scaled score^c^	100	17	107.1 ± 2.3	25	104.6 ± 1.9	25	110.7 ± 1.9	0.08
L-N sequencing scaled score^c^	10	14	11.1 ± 0.7	25	12.1 ± 0.6	24	10.2 ± 0.6	0.06
Digit span scaled score^c^	10	14	12.1 ± 0.9	25	10.9 ± 0.7	22	12.4 ± 0.7	0.27
CVLT short delay recall z-score^c^	0	14	0.6 ± 0.2	23	0.1 ± 0.2	24	−0.1 ± 0.2	0.07
CVLT long delay recall z-score^c^	0	14	0.7 ± 0.2^a,b^	23	0.0 ± 0.2	24	−0.1 ± 0.2	0.02*
CPT hit reaction t-score^d^	50	13	39.2 ± 2.5	25	38.8 ± 1.8	25	42.8 ± 1.8	0.27
CPT detectability t-score^d^	50	13	48.1 ± 2.7^b^	25	56.3 ± 1.9	25	53.3 ± 1.9	0.05*
UPSIT raw scores^c^	35+	19	23.6 ± 1.4^a,b^	25	31.0 ± 1.2	24	30.1 ± 1.2	<0.001*

*p* values shown for the main effect of group in univariate GLM analyses for each measure after correcting for age and gender

*significant at *p* < 0.05

^a^different from HC group

^b^different from T1DM group

^c^higher average = better performance

^d^lower average = better performance

#### Parent IQ estimate

The average parent WTAR verbal intelligence score did not differ across groups (F_2, 49_ = 1.31, *p* = 0.28).

#### Verbal and spatial intelligence

There was no main effect of group for VIQ (F_2, 59_ = 0.36, *p* = 0.70) or SR standard score (F_2, 64_ = 2.63, *p* = 0.08). On a trend level, HC performed better on SR than T1DM. WFS did not differ from either T1DM or HC.

#### Memory

Groups differed at a trend level on LNS (F_2, 59_ = 2.94, *p* = 0.06; T1DM > WFS > HC), but did not differ on DS (F_2, 57_ = 1.33, *p* = 0.27). Groups also differed at a trend level on CVLT short-term memory (F_2, 58_ = 2.72, *p* = 0.07; WFS > T1DM > HC). In addition, there was a main effect of group on CVLT long-term memory (F_2, 58_ = 4.01, *p* = 0.02), with the WFS group performing better (remembering more words after a 20 min delay) compared to both the T1DM (*p* = 0.02) and HC groups (*p* = 0.01).

#### Attention and impulsivity

Groups did not differ on CPT Hit Reaction Time (F_2, 60_ = 1.33, *p* = 0.27). However, there was a main effect of group on CPT Detectability (F_2, 60_ = 3.15, *p* = 0.05) with the WFS group showing better attentiveness and lower rates of impulsivity compared to the T1DM group (*p* = 0.02), but not to HC group (*p* = 0.12).

#### Smell identification

**Fig. 1:**
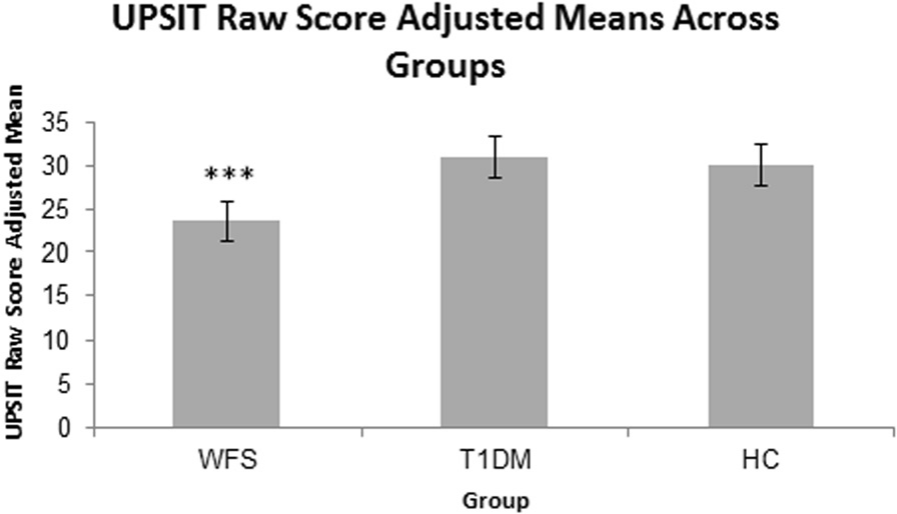
UPSIT raw score differed across groups after correcting for age and gender (F_2, 63_ = 9.38, ***p < 0.001). The WFS group performed significantly worse on the UPSIT than the T1DM group (*p* < 0.001) or the HC group (*p* < 0.001)

Groups differed on UPSIT raw score (F_2, 63_ = 9.38, *p* < 0.001), with the WFS group scoring lower than both the T1DM (*p* < 0.001) and HC groups (*p* < 0.001) (Fig. 1). Upon reviewing the UPSIT raw score data, we identified 3 older WFS patients that were much more impaired than other patients. We analyzed the data excluding these 3 WFS patients and still found group differences (F_2, 60_ = 7.52, *p* < 0.001), with the WFS group scoring lower than the T1DM (*p* < 0.001) and HC (*p* < 0.001) groups.

### Psychiatric and behavioral interview and assessments

#### Psychiatric interview

Nineteen WFS patients completed a psychiatric interview. Four WFS patients were currently prescribed psychoactive medications. Three patients were prescribed SSRIs (duloxetine, fluoxetine, sertraline) for anxiety and/or depression, and 1 of those patients was also prescribed lisdexamfetamine and atomoxetine for ADHD. The patient on fluoxetine was also taking risperidone for bipolar disorder. One patient was only taking lorazepam, a benzodiazepine, for anxiety symptoms. Three WFS patients reported a previous psychiatric diagnosis (diagnoses included: Generalized Anxiety, Major Depressive Disorder, ADHD, OCD, Bipolar Disorder), and 2 WFS patients reported undiagnosed symptoms, including anxiety, depression, and OCD. Four WFS patients were classified as having no psychiatric disorder by the study psychiatrist. One WFS patient was not fully evaluated due to incomplete psychiatric inventory questionnaires. The study psychiatrist assigned 10 patients a “best estimate diagnosis” of Unspecified Anxiety Disorder. Of those 10 patients, six received a co-morbid diagnosis of at least one other disorder, including Autism Spectrum Disorder, Major Depressive Disorder, ODD, OCD, and/or ADHD. Six WFS patients (patients were 13 years old or older) received a diagnosis of Hypersomnolence Disorder, and all of these patients had at least one other diagnosis. Other “best estimate diagnoses” occurring in those without a diagnosis of Unspecified Anxiety Disorder included a diagnosis of Unspecified Bipolar Disorder (*n* = 1), Separation Anxiety (*n* = 1), and Specific Phobia (*n* = 1).

#### Psychiatric inventories

Of the 19 WFS patients who were clinically assessed, 12 WFS, 21 T1DM, and 20 HC completed the parent-reported CASI-4R. Eight WFS, 13 T1DM, and 16 HC completed self-reports. The psychiatric inventory data only included English-speaking WFS patients. Four WFS patients were missing at least one parent or self-report instrument. Two of these patients did not have either report. One HC participant was not included because age was outside the age range for both the CASI-4R and YI-4R.

#### Neuro-developmental and disruptive behavior disorders

16.7 % WFS, 4.8 % T1DM, and 5.0 % HC met criteria for one or more neuro-developmental or disruptive behavior disorder on the parent-report. 7.7 % T1DM and 12.5 % HC met criteria on the self-report. None of the WFS patients self-endorsed having neuro-developmental or disruptive behavior symptoms above threshold. Groups did not differ on how likely they were to meet the criteria for a neuro-developmental or disruptive behavior disorder on the parent- or self-report (*χ*^2^ (*N* = 53) = 1.85, *p* = 0.40; (*χ*^2^ (*N* = 37) = 1.12, *p* = 0.57).

#### Anxiety disorders

50.0 % WFS, 38.1 % T1DM, and 30.0 % HC met criteria for one or more anxiety disorder on the parent-report. 12.5 % WFS, 15.4 % T1DM and 25.0 % HC met criteria on the self-report. Groups did not differ on how likely they were to meet the criteria for an anxiety disorder on the parent- or self-report (*χ*^2^ (*N* = 53) = 1.28, *p* = 0.52; (*χ*^2^ (*N* = 37) = 0.71, *p* = 0.70).

#### Mood disorders

8.3 % WFS, 4.8 % T1DM, and 0 % HC met criteria for one or more mood disorder on the parent-report. 12.5 % WFS, 7.7 % T1DM, and 6.3 % HC met criteria on the self-report. Groups did not differ on how likely they were to meet the criteria for a mood disorder on the parent- or self-report (*χ*^2^ (*N* = 53) = 1.52, *p* = 0.47; (*χ*^2^ (*N* = 37) = 0.28, *p* = 0.87).

#### Sleep questionnaires

Table 4 reports group means and standard errors for the PSQ measure. There was a main effect of group on the parent-reported PSQ total score (F_2, 49_ = 7.55, *p* < 0.001), with more symptoms reported for the WFS group than the T1DM (*p* = 0.02) and HC groups (*p* < 0.001). Groups also differed on the PSQ sleepiness subscore (F_2, 49_ = 4.98, *p* = 0.01), again with the WFS group having more symptoms than the other two groups (T1DM, *p* = 0.03; HC, *p* = 0.01). Groups did not differ in either the PSQ snoring (F_2, 49_ = 0.49, *p* = 0.62) or PSQ behavior (F_2, 49_ = 1.91, *p* = 0.16) subscores. Groups did differ in how likely they were to fall in the ‘at-risk for sleep problems’ range on the PSQ sleepiness score (*χ*^2^ (*N* = 54) = 12.63, *p* < 0.01; 71 % WFS, 24 % T1DM, 16 % HC), but not in the PSQ total score (*χ*^2^ (*N* = 54) = 1.82, *p* = 0.40; 7 % WFS, 10 % T1DM, 0 % HC), PSQ snoring score (*χ*^2^ (*N* = 54) = 1.82, *p* = 0.40; 7 % WFS, 10 % T1DM, 0 % HC), or PSQ behavior score (*χ*^2^ (*N* = 54) = 3.26, *p* = 0.20; 0 % WFS, 10 % T1DM, 0 % HC).

**Table 4:** Sleep measures adjusted means (±S.E.)

	Normative average	WFS		T1DM		HC		*p*
		*N*	*M*	*N*	*M*	*N*	*M*	
PSQ total score^c^		14	0.21 ± 0.0^a,b^	21	0.12 ± 0.0	19	0.06 ± 0.0	<0.001*
PSQ sleepiness^c^		14	0.48 ± 0.1^a,b^	21	0.21 ± 0.01	19	0.16 ± 0.1	0.01*
PSQ snoring^c^		14	0.12 ± 0.1	21	0.10 ± 0.0	19	0.05 ± 0.1	0.62
PSQ behavior^c^		14	0.07 ± 0.0	21	0.10 ± 0.0	19	0.03 ± 0.0	0.16

*p* values shown for the main effect of group in univariate GLM analyses for each measure after correcting for age and gender

*significant at *p* < 0.05

^a^different from HC group

^b^different from T1DM group

^c^lower average = better sleep

In a separate analysis of PSQ total score, older WFS patients were removed in order to reflect the age range intended for this questionnaire. Groups still differed on PSQ total score (F_2, 46_ = 9.11, *p* < 0.001), with the WFS group reporting more symptoms than the T1DM (*p* = 0.04) and HC groups (*p* < 0.001).

### Correlations

**Fig. 2:**
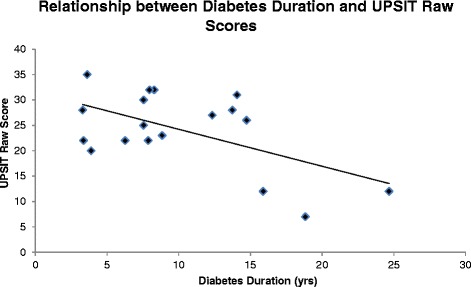
There was a significant relationship between diabetes duration and UPSIT raw scores in the WFS group (*r* = −0.56, *p* = 0.02), indicating that smell identification decreased with increased exposure to diabetes

Within the WFS group, UPSIT raw scores correlated with WFS diabetes duration (*r* = −0.56, *p* = 0.02) such that longer duration was associated with lower smell identification performance (Fig. 2). UPSIT raw scores did not correlate with age or the WURS total or subscale scores. All other variables that were different across groups (CVLT long-delay recall, CPT Detectability, PSQ total score, PSQ sleepiness) did not correlate with WURS or diabetes duration.

## Discussion

The purpose of this study was to determine whether there are any cognitive or psychiatric symptoms in relatively early stage WFS. This study advances the literature by directly assessing both WFS patients and age- and gender-matched control groups with standardized, quantitative assessments. Overall, we found selective deficits in WFS patients in smell identification and sleep disturbances, but no impairment in cognitive performance. Based upon a clinical evaluation of WFS patients only, we found that psychiatric and behavioral problems were present and consisted primarily of anxiety and hypersomnolence. However, when self- and parent-reported psychiatric symptom inventories were compared to control groups, there was no difference in rates of these reported symptoms. Smell identification deficits were associated with diabetes duration, but there were no other relationships between proxies of disease severity and assessment results. These results indicate that in relatively early stage WFS, smell tests and sleep questionnaires distinguish WFS from control groups better than cognitive tests or self- or parent-reported psychiatric questionnaires. However, psychiatric interviews of WFS patients suggest that there are diagnosable psychiatric conditions that in some cases are well-managed with pharmacological treatment. Although it is not clear if the rate of diagnosable psychiatric conditions in WFS differ from control groups from this study, longitudinal interview data in our WFS cohort may determine if psychiatric diagnoses are a useful index of disease progression.

Past studies have reported either cognitive impairment (learning difficulties, memory issues, decreased verbal performance) [12–15] or normal cognitive development in WFS patients [17, 18]. However, many of these studies were case reports without quantitative data on cognitive measures, had very small sample sizes, or were based on clinical impressions of older, more affected patients. In contrast, using structured testing and age- and gender-matched control groups, we found that WFS patients perform similarly to and in some cases better than controls, suggesting that cognitive impairment is not an early or prominent feature of WFS. It is possible, however, that cognitive impairment may evolve in the later stages of the disease. Interestingly, the WFS group performed better on a few tests, which could be due to higher motivation, perhaps to compensate for hearing or vision loss, or because of practice effects, as some had performed these tests in previous years. However, we found that a smaller subset of these WFS patients performed within or above the average range for verbal intelligence, memory, and attention on their first year of clinic [8], suggesting that even without practice they are performing well. The issues of practice effects or later degeneration of higher order cognitive function are best addressed with longitudinal data from both WFS and controls.

Similar to the cognitive data, our psychiatric and behavioral data yielded results somewhat inconsistent with past literature. Previous reports have indicated that there may be a role of the *WFS1* gene in depression and suicide attempts [35, 36], as carriers of one *WFS1* mutation had a greater risk for lifetime psychiatric symptoms. Studies of WFS patients have also suggested that WFS patients suffer from high rates of depression and anxiety [14, 15, 37]. However, these reports were based on overall clinical impressions and self-report of diagnoses rather than standardized measures or structured clinical interviews and lacked appropriate control groups. Notably, during a clinical interview with the study psychiatrist, many of the WFS patients reported previous symptoms consistent with an Unspecified Anxiety Disorder. However, in more than one case, the study psychiatrist noted that the patient’s parent was very concerned about the patient’s anxiety and psychiatric symptoms, but the patient did not agree they had significant symptoms. There is a possibility that this could be influenced by parental anxiety about their child’s symptoms, or it could be due to limited insight of the patient. Furthermore, we did not find elevated psychiatric symptoms in the standardized parent- or self-report of current symptoms in three critical psychiatric domains—Neuro-developmental and Disruptive Behavior, Anxiety, and Mood disorders—as compared to the control groups. The apparent incongruity of results between our findings and previous reports could indicate: (1) younger WFS patients may not experience the same severity of psychiatric issues as older, more impaired WFS patients; (2) that our patients have received appropriate support and treatment for their symptoms; or (3) these standardized assessments did not detect clinically relevant symptoms for this particular cohort of patients. Nevertheless, the psychiatric symptoms the WFS patients may experience are generally treatable and manageable as indicated by the lack of self-reported symptoms.

Consistent with past reports [17, 15, 38, 39], we saw selective deficits in WFS patients in smell identification. Olfactory dysfunction often is an early indicator of neurodegenerative diseases, such as multiple sclerosis (MS), Parkinson’s disease, and Alzheimer’s disease [40]. Several WFS studies have reported a range of olfactory issues in patients, including anosmia [17, 15, 38, 39]. However, these studies did not detail how they assessed patients, did not include control groups, and were primarily case reports of older adults. Our results indicate that smell identification deficits are present early in WFS, even compared to a T1DM control group, and are not explained by differences in cognitive function. Further, since smell deficits were worse in those with greater duration of diabetes, it is possible that smell may be a potential marker of disease progress; however, this hypothesis will need longitudinal data to confirm.

Sleep dysfunction was also common in our WFS group. WFS patients and their parents reported more sleep-related problems, such as snoring, heavy breathing, bed wetting, and sleepiness than controls. Furthermore, the study psychiatrist found that the majority of adolescent and adult (6/10 patients ≥ 13 years of age) WFS patients qualified for hypersomnolence disorder. It is unclear whether these sleep problems are central to WFS or due to the presence of a chronic disease like type 1 diabetes or diabetes insipidus, which can increase the frequency of nocturnal urination and sleep disruption. Sleep problems are prevalent among individuals with type 1 diabetes [41–43]. Matyka et al. found that compared to unaffected children, children with T1DM woke up more during the night and slept less. However, our T1DM and control groups did not differ in sleep problems, so it is unlikely that diabetes per se explains sleep problems in our WFS sample. Sleep studies using actigraphy to record motion and pulse oximetry to monitor respiration could help distinguish between the mitigating clinical and environmental factors altering sleep behavior in WFS and possible underlying neurologic dysfunction. Given that death due to WFS has been ascribed to sleep apnea in some cases [11], these features deserve closer attention and evidence-based intervention.

The pattern of spared and affected non-motor neurological function that we describe in this paper is generally consistent with the known pattern of structural brain abnormalities in WFS. We have observed striking brain volume differences in lower brain order systems like the brainstem and cerebellum in WFS (including some of the patients represented in this paper), but few differences in cortical volume measurements [8]. Smell and sleep are mediated by complex interactions between the brain stem, limbic system, hypothalamus, and some cortical regions [40, 44, 45], whereas cognitive function relies primarily on higher order cortical networks. Further cross-sectional and longitudinal structure-function analyses will be necessary to determine whether these relationships exist in WFS.

Our study was a thorough examination of the cognitive, psychiatric, and behavioral outcomes seen in WFS. The major strengths of the study are the inclusion of an age- and gender-matched control group and the use of standardized measures. However, there were some limitations to the study. First, although our WFS group and control groups performed similarly on the cognitive tasks, we may have selected a WFS cohort that was a high functioning group with access to more resources than the average WFS family. However, a wide range of symptom severity was represented in our WFS group, suggesting that they were not high functioning in all domains. Also, this may be a function of the wide age range in our WFS patients, from early childhood to early adulthood. This age range could also obscure the possibility that psychiatric symptoms and diagnoses are a later emerging issue in WFS. Although we saw no obvious relationship between age and symptoms, aside from a possibly higher rate of hypersomnolence in adolescence and adulthood, our sample is likely too small to detect such a pattern. Second, some of our WFS patients were exposed to the testing measures in previous clinics, while the control groups had not been exposed. However, regardless of practice, our WFS patients still showed significant impairments in smell identification. Third, the WFS group and control groups did not receive the same psychiatric evaluation, and the control group was screened for psychiatric diagnoses and medication use prior to study enrollment. Four WFS patients were currently prescribed psychoactive medication for psychiatric symptoms. These patients may have psychiatric symptoms that were masked by medication use, leading to decreased reporting of symptoms on the psychiatric inventories. Furthermore, we cannot draw conclusions about differential rates of psychiatric diagnosis between the groups due to the exclusion criteria for the control groups. Based upon the discrepancies in psychiatric evaluation, parent-report and self-report psychiatric symptoms are clearly complicated in WFS, and it may be difficult to distinguish what symptoms are related to pathological WFS versus symptoms related to having a severe chronic and degenerative illness with multisensory system effects. Future studies on psychiatric illness in WFS could explore different self-report measures than the ones used in the current study. Nonetheless, these findings indicate that some psychiatric symptoms may be manageable in WFS patients with typical pharmacologic treatments.

## Conclusion

The results of this study indicate that cognitive performance and self- and parent-reported psychiatric symptoms are not different from controls in the early stages of WFS, while smell and sleep abnormalities manifest throughout the age range. This pattern may differ over time, as neurodegenerative processes continue, and thus, in later stages of the disease cognitive and psychiatric symptoms may play a larger role in WFS. Although self-reported psychiatric measures and clinical evaluations yielded incongruent results, at the present time, patients did not report significant impairment from active psychiatric symptoms. Given the potential impact of all of these domains on quality of life, symptomatic treatments (e.g. anti-depressants) and interventions for the fundamental neurodegenerative process are of great interest. Longitudinal assessments of these domains are currently underway, which will clarify whether cognitive and psychiatric disorders are later emerging problems in WFS and will identify measures that might serve as biomarkers of disease progression for use in future clinical trials.
